# Developing an Intervention to Improve Sexual Health Assessment and Care in Men With Inflammatory Bowel Disease

**DOI:** 10.1111/jan.70199

**Published:** 2025-09-05

**Authors:** Sara Ma, Greg Forshaw, Mona Kanaan, Peter Knapp, Wayne Robinson, Christian Selinger, Paul Galdas

**Affiliations:** ^1^ School of Science, Technology and Health York St John University York UK; ^2^ York and Scarborough Teaching Hospitals, NHS Foundation Trust York UK; ^3^ Department of Health Sciences University of York York UK; ^4^ Hull and York Medical School University of York York UK; ^5^ Patient and Public Representative UK; ^6^ St James University Hospital, The Leeds Teaching Hospitals NHS Trust Leeds UK

**Keywords:** chronic illness, clinical nurse specialist, gastroenterology, men's health, qualitative approaches, research methods, sexual health, survey designs

## Abstract

**Aim:**

To co‐produce a prototype intervention to help nurses improve the assessment and care of the sexual health needs of men with inflammatory bowel disease.

**Background:**

Inflammatory bowel disease can have a significant impact on the sexual health and well‐being of men, but has largely been neglected in research and clinical guidelines. Men with the disease report that sexual health is not discussed during consultations, while healthcare practitioners describe a lack of confidence to initiate sexual health assessments. At present, no evidence‐based tool exists to support nurses in detecting, assessing, and providing care for the sexual health of men with the disease.

**Design:**

A mixed‐methods study shaped by phase 1 of the Medical Research Council's framework for the development of complex interventions.

**Methods:**

(1) Cross‐sectional surveys of (i) men with inflammatory bowel disease, (ii) nurses, and (iii) inflammatory bowel disease services to determine the current state of sexual health provision across the UK National Health Service. (2) Semi‐structured interviews with men and the partners of men with IBD and asynchronous focus groups with health professionals to explore appropriate and acceptable ways to provide sexual healthcare. (3) Three consecutive co‐production workshops inclusive of men with the disease, healthcare professionals, and stakeholders to formulate a prototype intervention.

**Implications for the Profession and/or Patient Care:**

This study will create an evidence‐based prototype intervention that will provide nurses with the knowledge and skills required to effectively assess the sexual health needs of men with inflammatory bowel disease and provide appropriate, patient‐centred care.

**Patient Contribution:**

The study design was supported by a patient group. The study delivery will be supported by a patient co‐investigator and stakeholder group inclusive of men with lived experience of the disease.

**Reporting Method:**

This report adheres to the SPIRIT 2013 checklist for standard protocol items for clinical trials.

**Trial Registration:**

clinicaltrials.gov ID: NCT06562751

**Summary**

- What is already known?
  ○ Nurses often lack the confidence to address the sexual health needs of their patients.
  ○ The sexual healthcare needs of men with inflammatory bowel disease are under‐researched.
  ○ There is a lack of disease‐specific healthcare information, training and guidance on sexual healthcare.
- What this paper adds?
  ○ A protocol for a research study to develop a nursing‐focused, evidence‐based prototype intervention.
  ○ Mixed methods, collaborative study design that involves co‐production methods with meaningful public and patient involvement.
  ○ Transparency of study design to support future nursing research.
- Implications for practice/policy
  ○ Evidence to inform nursing practice in the sexual health assessment and support of men with inflammatory bowel disease.
  ○ Awareness of nurse‐led, co‐produced research that supports evidence‐based and feasible clinical interventions.
  ○ Promotion of holistic, disease‐ and gender‐considered sexual healthcare.

## 1 Introduction

Inflammatory bowel disease (IBD) is a chronic, relapsing–remitting condition that can induce diarrhoea, rectal bleeding, abdominal pain, malnutrition, and fatigue. Symptoms are not always confined to the gastrointestinal tract and 25% of patients experience at least one extra‐intestinal manifestation of the disease in the joints, skin, or eyes (Kilic et al. 2023). Globally, there are over 5 million people living with IBD (Wang et al. 2023) with the UK population prevalence estimated to be 1 in 123, or approximately 540,000 people (Crohn's and Colitis UK 2022). The disease is broadly classified into two main subtypes: ulcerative colitis and Crohn's disease, each characterised by distinct pathological and clinical features. IBD poses a significant challenge to individuals in regard to their physical, psychological, and social well‐being. When severe, patients may be hospitalised and require surgery. The peak onset of the disease is widely reported as being between 15 and 30 years of age, a period during which people develop their sexual and personal identities.

Sexual health is defined as a state of physical, emotional, mental, and social well‐being in relation to sexuality, and is an important determinant of quality of life (World Health Organisation 2000). Sexual health problems can adversely impact overall well‐being at any stage of adolescence or adulthood, affecting both individuals in established relationships and those who are single and seeking to engage in sexual activity (Hinchliff et al. 2023).

There is increasing recognition that IBD can substantially affect sexual functioning, with both men and women carrying an increased risk of sexual dysfunction (Chen et al. 2022; Zhao et al. 2019). The unpredictable and chronic nature of the disease combined with its embarrassing effects on the body (e.g., altered bowel patterns, perianal disease, stomas, and changes in body physique) can induce disease‐specific sexual health issues (Moya et al. 2024). Variations in anatomy, physiology, and psychology mean the disease produces varying sexual health challenges and needs in men and women (Maunder et al. 1999). Recognising and addressing the sexual health needs of people based on biological and gender differences is a key objective of the WHO's Action Plan for Sexual and Reproductive Health (World Health Organisation 2016). While many studies have addressed the sexual health needs of women with IBD (including sexuality, pregnancy, and fertility), insufficient attention has been paid to the needs of men (Ma et al. 2020; Allocca et al. 2018).

Evidence suggests that in the UK, men's sexual health concerns are rarely considered during routine IBD care and men report that they are not provided with sexual health information or support (Ma et al. 2024). The European Crohn's and Colitis Organisation (ECCO) recommends that IBD nurses assess their patients' sexual health concerns during routine care (Kemp et al. 2018) but there is a lack of disease‐specific guidance on how to do this, with nurses from across the globe, including Japan, Australia and Canada, identifying a lack of education and confidence to address sexual health concerns in IBD (Wakai et al. 2025). This research addresses the need for an intervention that will support nurses to identify the sexual health needs of men with IBD and subsequently co‐ordinate and provide specialist care.

## 2 Background

### 2.1 Men's Sexual Health in IBD

Reported rates of sexual dysfunction in men with IBD range from 10% to 50% (O'Toole et al. 2014) and men with IBD have a 41% higher risk of sexual dysfunction as compared to men without IBD (Zhao et al. 2019). The assessment of erectile function has been the primary focus of sexual health assessment and research in men with IBD (Ma et al. 2020). While erectile dysfunction is a recognised post‐operative complication for some IBD‐related surgeries, men with IBD were more likely to obtain a prescription for erectile dysfunction (ED) than men without IBD regardless of surgery history (Friedman et al. 2018). Additionally, while the risk of ED generally increases with age, men with IBD are affected at a younger age compared to the general population (Kao et al. 2016).

Recent research has revealed that although ED is an important consideration for men with the disease, the influence of IBD on the wider aspects of sexual well‐being such as intimate partnerships, self‐perception, and expressions of masculinity holds a greater significance for men (Ma et al. 2024). Importantly, half of sexually inactive men with IBD attribute abstinence from intercourse to their underlying disease (O'Toole et al. 2014).

Gay, bisexual, and transgender men with IBD appear particularly disadvantaged in accessing healthcare due to a fear of judgement, lack of inclusion of same‐sex partners in healthcare interactions, a reported lack of understanding from healthcare practitioners regarding sexual practices, and the absence of information on the safety of anal sex in active disease (Ma et al. 2024; Dibley et al. 2014). In order to effectively support this patient group, specific information about gay and bisexual sexual activity when living with IBD is required (Dibley et al. 2014).

### 2.2 Nursing Provision of Sexual Health to Men With IBD

IBD specialist nurses hold a pivotal role in IBD patient care and provide rapid access to disease management, therapy monitoring, psychosocial support, and continuity of care (Lamb et al. 2019). At the point of registration, UK nurses are expected to be able to discuss sexual behaviours and practices (Nursing and Midwifery Council 2018), yet it is widely acknowledged that discussion of sexual health concerns is a challenge across a range of long‐term conditions (McGrath et al. 2021). While there is limited evidence in IBD specifically, nurses working in long‐term conditions report lacking the knowledge, time, and confidence to address the psychological, social, and sexual aspects of their patients' illness (O'Connor et al. 2019).

Little is known about the practice patterns of IBD nurses regarding sexual health and there is scant evidence to guide nursing care in this important aspect of patient well‐being (Ma et al. 2020). People with IBD have reported feeling discouraged from discussing sexual health due to a perceived lack of time, clinician interest, and informational resources (Fourie et al. 2021). The challenge of initiating sexual health discussions is likely compounded for men as masculine gender norms can create additional barriers to seeking and receiving adequate sexual healthcare (Persson et al. 2022).

Evidence suggests that men with long‐term conditions may benefit from models of service delivery that are tailored to their gender‐specific needs and preferences (Galdas et al. 2014). Global health organisations have called for the development of gender‐transformative interventions for men and boys to improve sexual and reproductive health and rights for all (United Nations Fund for Population Activities 2023; World Health Organisation 2016). Gender‐transformative interventions seek to challenge gender inequality by transforming harmful gender norms, roles, and relations through the inclusion of strategies to foster progressive changes in power relationships between women and men (Ruane‐McAteer et al. 2020).

This research will be guided by the 5C (co‐production, cost, context, content and communication) framework for developing gender‐transformative men's health programmes (Galdas et al. 2023) to ensure the development of a gender‐sensitised intervention that will support nurses to assess and care for men's sexual health, which could improve overall quality of life (Barnhoorn et al. 2022; Allocca et al. 2018; Galdas et al. 2014).

## 3 The Study

### 3.1 Key Research Questions

- What is the current state of National Health Service (NHS) provision of sexual health assessment and care to men with IBD?
- What are the barriers and facilitators to specialist nurses' assessment of the sexual health needs of men with IBD?
- What type of interventions are likely to be effective and acceptable in improving nurse‐led sexual health assessment and care of men with IBD?

### 3.2 Aim

To co‐produce a prototype intervention that will support nurses to detect, assess, and provide care for the sexual health and well‐being of men with IBD.

### 3.3 Objectives

- Identify how the sexual health of men with IBD is currently assessed and cared for by specialist nurses in the NHS.
- Gather ideas on appropriate and acceptable ways in which men's sexual health care in IBD could be improved.
- Co‐produce a prototype intervention to improve the assessment and care of the sexual health needs of men with IBD.

### 3.4 Study Design

**FIGURE 1:**
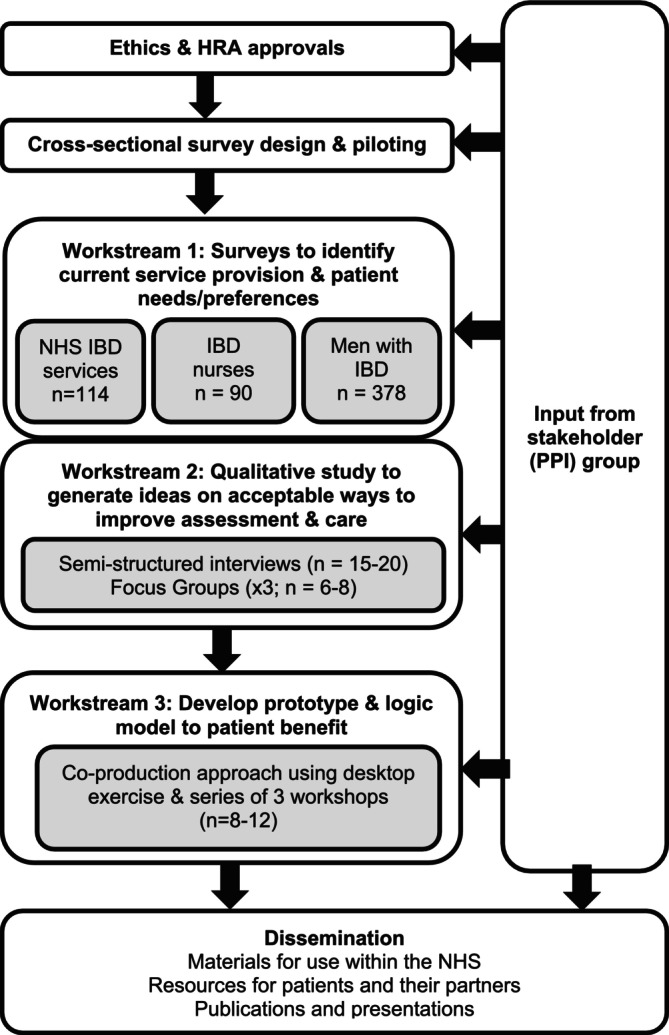
Study flow chart.

This is a mixed‐methods study involving three workstreams (Figure 1) that align with the research objectives of phase 1 of the revised Medical Research Council (MRC) framework for the development and evaluation of complex interventions (Skivington et al. 2021).

Workstream 1 will consist of three national cross‐sectional surveys to assess current sexual health practices and services. Survey 1 will assess adult NHS IBD services in the UK to evaluate the availability of sexual health provisions. Survey 2 will target UK‐based IBD nurses to examine current training, practices, and service delivery related to sexual health. Survey 3 will gather insights from men living with IBD in the UK, exploring their experiences of sexual health provision, alongside their perceived needs and preferences for care.

Workstream 2 will run concurrently with Workstream 1 and involve semi‐structured interviews with men with IBD and their partners. Additionally, online asynchronous focus groups will be held with healthcare practitioners who provide healthcare to men with IBD, including doctors, nurses, and clinical psychologists. This arm of the study has two aims: to identify factors influencing sexual health assessment and care, and to generate ideas for appropriate and acceptable improvements in service delivery.

Workstream 3 will use a co‐production approach (Hawkins et al. 2017) to integrate findings from Workstreams 1 and 2. A desktop exercise will compare, contrast, and qualitatively synthesise data from Workstreams 1 and 2 to map barriers and facilitators to sexual health assessment onto the Capability, Opportunity, and Motivation‐Model of Behaviour (COM‐B) (Michie et al. 2011). The resulting summary report will outline key findings and propose candidate interventions designed to improve nurse‐led sexual health assessment and care for men with IBD. Subsequently, a series of three workshops (Table 1) will iteratively develop the prototype intervention using a range of co‐production tools and techniques, including brainstorming, mind mapping, and group discussion. Particular emphasis will be placed on the intervention's context, content, and communication strategies, whilst working to transform gender norms and relations that can harm sexual health (e.g., ideas of “manhood” that are predicated on taking risks and not seeking help) to promote positive health changes for men and their partners.

**TABLE 1:** Description of co‐production workshops.

Workshop 1	The co‐production process will be introduced, and participants will review the initial list of candidate interventions. Group discussions and consensus‐building will be used to agree on an intervention structure
Workshop 2	Participants will discuss the intervention's mechanisms, focusing on design, content, and usability
Workshop 3	A final iteration of the prototype intervention will be shared for discussion, with emphasis on refining and identifying effective delivery mechanisms

### 3.5 Recruitment and Sampling

#### 3.5.1 Eligibility

**FIGURE 2:**
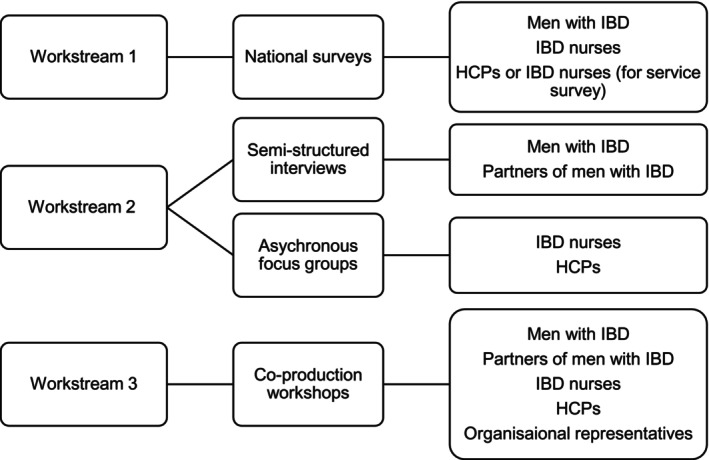
Workstreams and participant groups.

The study will include the following groups of participants (see Figure 2):

1. **Men with a diagnosis of IBD**: individuals who identify as male, are aged 18 years or above, and have been diagnosed with IBD, including Crohn's disease, ulcerative colitis, or IBD‐unclassified.
2. **Partners of men with IBD**: individuals who are currently or have previously been in an intimate partnership with a person identifying as male and diagnosed with IBD.
3. **Registered nurses**: nurses whose current role involves providing care to patients with IBD.
4. **Healthcare professionals**: professionals registered with the General Medical Council (GMC), Nursing and Midwifery Council (NMC), or Health and Care Professionals Council (HCPC), who currently or have previously provided care to men diagnosed with IBD.
5. **Organisational representatives**: representatives from charitable, not‐for‐profit, professional, public, or governmental organisations involved in supporting men with long‐term conditions or IBD.

All participants must be aged 18 years or older, fluent in English, and able to provide informed consent to take part.

#### 3.5.2 Sample Size

##### 3.5.2.1 Survey of NHS IBD Services

The target is 114, representing 50% of the 228 known IBD services identified in 2019 IBD audit (Inflammatory Bowel Disease United Kingdom 2021). This sample size was calculated to allow key binary indicators to be estimated with a margin of error (MoE) of 6.5% within a 95% confidence interval (CI).

##### 3.5.2.2 Survey of IBD Nurses

The target sample size is 90 participants, representing 30% of the estimated 300 IBD nurses in the UK (Leary et al. 2018). This sample size will enable key binary indicators to be estimated with a MoE of 8.7% for a 95% CI. A more conservative proportion of the nursing population was selected due to the challenges faced in recruiting busy NHS staff.

##### 3.5.2.3 Survey of Men With IBD

The target sample size is 378 male respondents, which would enable an estimate of the proportion of IBD patients who have had their sexual needs discussed with a MoE of 5% for a 95% CI. The sample calculation was informed by estimates from the IBD‐UK 2019 survey that was completed by approximately 3236 adult male respondents over a 7‐month period (Inflammatory Bowel Disease United Kingdom 2021).

##### 3.5.2.4 Semi‐Structured Interviews

The target sample size is 15 to 20 participants to enable a heterogenous sample sufficient to address the research questions to the point of data saturation (Hennink et al. 2017) while capturing variation in disease presentation and participant demographics, including age, race, sexual orientation, and relationship status.

##### 3.5.2.5 Asynchronous Focus Groups

Each group will involve 6–8 participants, with a total of three independent groups. This number will allow for diversity in professional groups and stakeholders while safeguarding effective facilitation.

##### 3.5.2.6 Co‐Production Workshops

Eight to twelve participants will participate in three sequential workshops. This number accommodates diversity among men with IBD, stakeholders, and health professionals, while ensuring that the workshops will yield tangible and actionable outputs.

#### 3.5.3 Participant Recruitment

**FIGURE 3:**
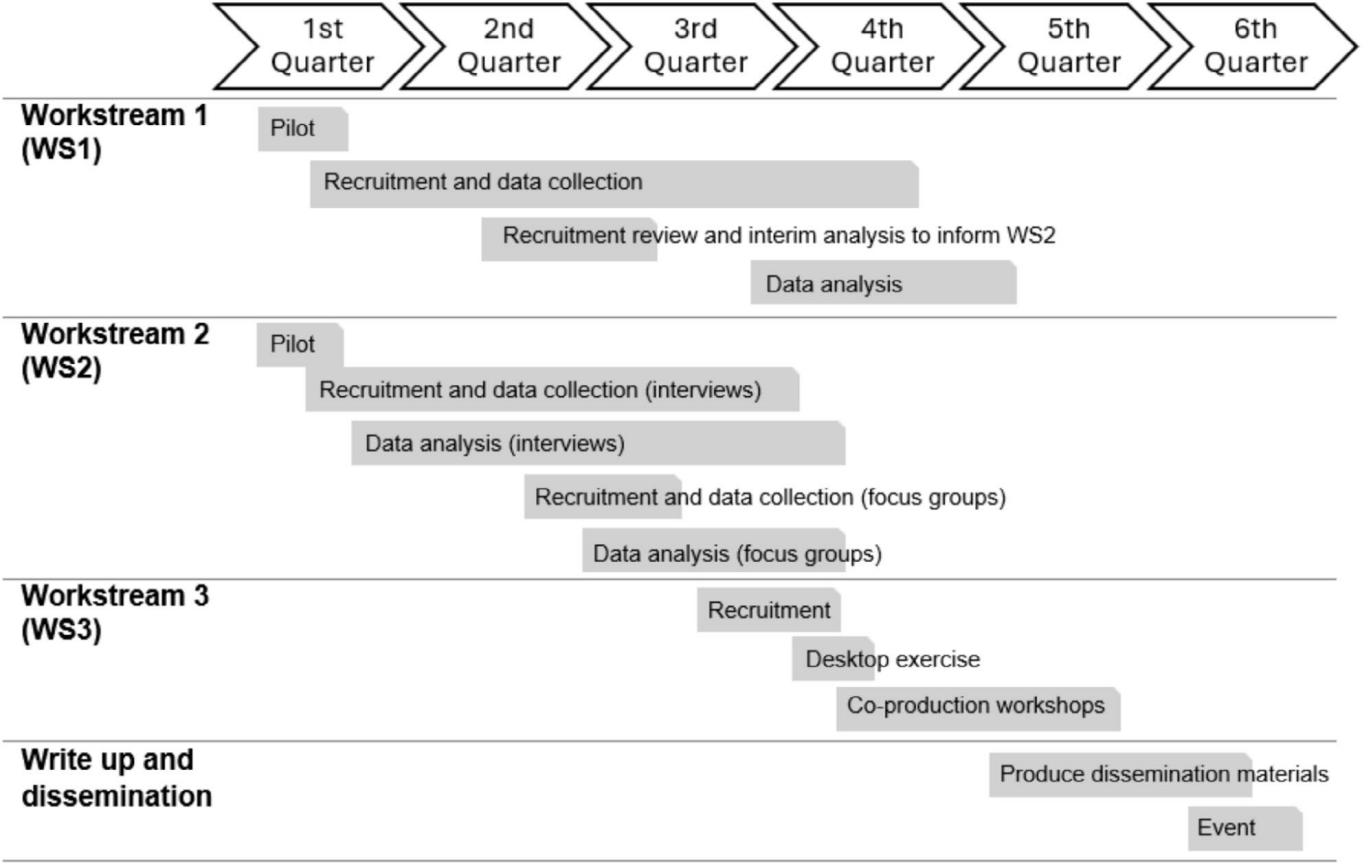
Study timeline.

The study timeframes including recruitment and enrolment of participants are presented in Figure 3. In Workstream 1, the survey of IBD services and nurses will adopt a census approach. The survey will be distributed to clinical leads and service managers across all NHS IBD services. For IBD nurses, the survey will be cascaded via clinical leads, the Royal College of Nurses IBD network, Crohn's and Colitis UK (CCUK), appropriate professional meetings and online advertisements. The survey of men with IBD will utilise targeted convenience sampling achieved by advertising on social media, as well as leaflets and posters disseminated through NHS sites, established patient networks and stakeholder organisations. To ensure adequate representation, recruitment efforts will focus on geographic regions with higher densities of under‐represented groups or populations less likely to engage with traditional or online methods of study advertising. The surveys will be open for 7 to 12 months depending on recruitment.

Survey respondents will have the option to express an interest in participating in Workstream 2. From these expressions of interest, a heterogenous sample of 15 to 20 men will be selected based on characteristics including age, ethnicity, partner status, sexuality, and IBD type. Similarly, participants for asynchronous focus groups will be selected using a combination of targeted recruitment and snowball sampling through professional colleagues. The focus groups and co‐production workshops will be promoted through professional networks to ensure participation from a wide range of stakeholders and professionals. Participants for the co‐production workshops will be identified through Workstreams 1 and 2, as well as through the stakeholder advisory group.

There will be no payment for participation in the surveys. However, a prize draw will be offered in the survey of IBD nurses (1× £50 high‐street voucher) and the survey of men with IBD (5× £50 high‐street vouchers). Those participating in interviews or focus groups will receive a £20 high‐street voucher for participation and compensated for reasonable expenses such as childcare.

### 3.6 Data Collection

Survey data will be collected using an online survey instrument (Qualtrics) and each survey should take an average of 15 min to complete. The survey of IBD services will cover several domains, including current sexual health assessment, care provision, availability of specialist nursing services, access to psychological and other support services, and provision of patient information. The survey of IBD nurses will assess current nursing practice and training available, as well as the perceived confidence in and barriers to delivering sexual health assessment, care, and advice to men with IBD. The survey of men with IBD will ask questions regarding sexual health experiences, care needs, and preferences (Table 2). Surveys were developed in collaboration with a public engagement group using themes identified through a prior scoping review and primary research (Ma et al. 2025, 2024, 2020). Surveys were piloted with men with IBD and healthcare professionals.

**TABLE 2:** Questions included in the survey of men with IBD.

Sexual health and experiences	Sexual healthcare needs and preferences
Which best describes you?^a^ (*In regards to your sexual experiences with other genders*) I struggle to get or maintain an erection^d^ IBD affects my desire for sex^d^ IBD prevents me having sex^d^ IBD prevents me having receptive anal sex or receiving anal stimulation ^d^ IBD medications that I have to administer rectally affect my sex life^d^ IBD medications that I take in my mouth or via injection affect my sex life^d^ Have any of the following symptoms and side effects of IBD ever negatively affected your sexual activities or satisfaction? *Rectal bleeding; Incontinence; Abdominal pain; Feeling unhygienic; Discharge from anus, rectum or back passage; Fatigue; Stoma; Perianal disease or fistulas, Oral symptoms such as mouth ulcers* ^d^	Sexual health should be discussed with all IBD patients^c^ Who should discuss sexual health with IBD patients?^a^ Please selected you preference (on gender of healthcare professional for discussions on sexual health)^a^ Please tell is when information and support should be provided^a^ In relation to receiving information and support for your sexual health and well‐being, please tell us how important are the following to you; *Overall sexual health and well‐being, advice specific to men, relationships, fertility, erectile function, surgery, managing faecal incontinence, stomas, perianal disease, receptive anal play and sex, gender identity and sexual orientation, diet and muscle mass, low mood, where to access support* ^f^ Please tell us how you would like sexual health and information, advice, and support to be provided^a^
Relationships	Body image
What is your marital or relationship status?^a^ IBD has affected me starting new (intimate or sexual) relationships^c^ IBD has contributed to relationship challenges^c^ I tell new or potential sexual partners about my disease^c^ I worry about what sexual partners will think about the disease^c^ My partner (or previous partners) have helped me to cope with my disease^c^ My partner (or previous partners) have helped me to manage my disease^c^	IBD makes it difficult to control my weight^c^ I struggle to maintain muscle mass because of my IBD^c^ IBD makes me feel less attractive^c^ Having a stoma negatively affects my body image^c^ Perianal disease negatively affects my body image^c^ Surgical scars negatively affect my body image^c^ Incontinence negatively affects my body image^c^
IBD characteristics and care	
How many NHS hospitals have been involved in the care of your IBD since diagnosis?^b^ In which NHS hospital have you most recently received care for your IBD?^a^ (*In regards to presentations and complications of IBD*, i.e., *perianal disease, fistulising disease, stoma etc*.) Do you have any of the following?^a^ In the last 3 months have your received any of the following treatments for your IBD?^a^ Do you have access to an IBD specialist nurse?^e^	f In the last 12 months have you had contact with an IBD doctor, surgeon or nurse?^e^ g In the last 12 months have you been in contact with an NHS dietitian in relation to your IBD?^e^ h In the last 12 months have you been in contact with a NHS clinical psychologist, counsellor or mental health advisor in relation to your IBD?^e^ i How well do you feel in regards to your IBD at the moment?^a^
Healthcare experiences	
I have discussed my overall sexual health and wellbeing with a member of my IBD team^e^ I have discussed my erectile function with a member of my IBD team^e^ I have previously trialled or currently take medications to support my erectile function (e.g., Viagra)^e^ In regards to screening for sexually transmitted infections or STIs (i.e., having a test to see whether you have an infection such as HIV, hepatitis, chlamydia, etc.) please tick all that apply;^a^	e I have discussed my sexual orientation with a member of my IBD team^e^ f I have discussed fertility or starting a family with a member of my IBD team^e^ g I have discussed body image with a member of my IBD team^e^ h I have had my sexual health formally assessed using a questionnaire or tool^e^ i I have been given written information on sexual health when living with IBD^e^ j I have brought a partner with me to an IBD healthcare appointment^e^
*Depending on the response of the above questions further questions open up asking whether the respondent is able to provide further information*.	
Questions about you	
How old are you?^b^ What was your sex assigned at birth? (*In regards to your sexuality*) Do you think of yourself as; ^a^ What type of Inflammatory Bowel Disease do you have?^b^ What age were you when first diagnosed with IBD?^a^ In regards to being or becoming a parent, please select one;^a^ Which best describes your employment?^a^	h Please select which of the following qualifications you have ever achieved^a^ i What is your total personal annual household income?^a^ j What ethnic group do you belong to?^a^ k Which option describes what you were doing in the last 7 days^a^ l What is your main language?^a^ m What is your religion?^a^
Key for multiple choice answers:	
Unique selection choice based on question. Numerical value range Strongly agree, agree, neutral, disagree, strong disagree, prefer not to say, not‐applicable	d Never, rarely, sometimes, often, always, prefer not to say, not applicable e Yes, no, unsure, prefer not to day f Very important, important, neutral, unimportant, very unimportant, does not apply to me

The individual semi‐structured interviews will gather in‐depth data on men's experiences of healthcare and perceptions on how healthcare practice could be improved. Men who are in a relationship will be offered the opportunity to invite their partner to an interview, which will be delivered in a conjoint or individual format according to participant preference. Single men and those who prefer not to involve their partner will be interviewed individually. Interview guides (Table 3) were developed using themes identified in preliminary public and patient involvement (PPI) work, in consultation with the study stakeholder group, and through prior research (Ma et al. 2024, 2020). Interviews will take place via videoconferencing or phone, according to participant preference. Interviews will be digitally audio‐recorded, transcribed and imported into NVivo (QSR International) qualitative data management software.

**TABLE 3:** Interview topic guide.

INTRODUCTION AND BACKGROUND
Recap aim of study and purpose of interview and core principles including confidentiality, safe‐space, how information will be used. *Ask participant to provide a short background including, age, sexual orientation, relationship status and information about their disease*.
TOPIC 1: SEXUAL HEALTH & WELL‐BEING
*Can you tell us how the sexual health of men with IBD might be affected?*
TOPIC 2: ASSESSMENT
*Thinking about interactions with IBD specialists, can you describe any experiences of being assessed for sexual health concerns?*
TOPIC 3: THE PATIENT‐PROFESSIONAL RELATIONSHIP
*Do you feel comfortable to ask your IBD clinicians about sexual health?*
TOPIC 4: TREATMENT
*Have you ever been offered any treatment or support for your sexual health?* *What else could be provided in terms of care for men with IBD for their sexual health and well‐being?*
TOPIC 5: INFORMATION
*Where might you go for information on sexual health when living with IBD?* *What information do you feel would be helpful to you?*
SUMMARY AND CLOSE DOWN
*Is there anything we have missed, or you would like to discuss further?* Provide support leaflet

*Note:* Only one or two example questions are included for each topic here; the full interview guide contains more example questions to be used.

Asynchronous focus groups, which are run online and allow participants to participate independently of one another, will be held with healthcare professionals. The groups will focus on 5 domains: (1) understanding current practices, (2) barriers to care, (3) clinical experiences, (4) making care more appropriate, and (5) needs and resources. The groups will be hosted on the Quallie (Collabito) platform, utilising discussion boards, live text chat, free‐text questions, and private diary sections. The study team will moderate using the comment function. The asynchronous approach will facilitate the scheduling of busy healthcare professionals and decrease the burden of participation. Data will be downloaded and imported into NVivo for analysis.

The co‐production workshops will take place using a hybrid format inclusive of face‐to‐face and communications via email or videoconferencing, supported with the asynchronous platform Quallie (Collabito). This will facilitate attendance from participants across the UK.

### 3.7 Data Analysis

The analysis of quantitative data will be descriptive, using appropriate summary statistics (frequencies/percentages for categorical variables; means/standard deviations or medians/quartile ranges for continuous variables, depending on their distribution). For key variables and scores, a 95% CI will be presented and differences between geographical regions evaluated. Several associations (including barriers to, and facilitators of, sexual health assessment for men) will be tested using logistic regression and estimates of the magnitude of association, for example, odds ratio, and corresponding 95% CI will be presented.

The analysis of qualitative data will be guided by Interpretive Descriptive methodology (Thorne 2016) and follow the six stages of thematic data analysis (Braun and Clarke 2006). In line with mixed‐methods approaches, qualitative and quantitative data will be integrated (Creswell and Clark 2017) to enable a comprehensive analysis and presentation of rich, relevant data necessary for developing an evidence‐based intervention during the co‐production workshops. As the survey data may continue to be received for a period beyond the start of the qualitative work, an interim data analysis will be conducted to allow the findings to inform and be integrated into this workstream. The interim analysis will also support ongoing recruitment and identify populations where targeted recruitment is needed.

### 3.8 Ethical and Regulatory Considerations

Ethical approval has been granted by an NHS Research Ethics Committee [IRAS 334340: Ref 24/EE/0158. CPMS: 58565]. The study was also approved by a research ethics committee York St. John University and a research advisory group at York and Scarborough Teaching Hospitals, NHS Foundation Trust, who are sponsoring the study.

Informed consent procedures will adhere to The International Conference on Harmonisation Good Clinical Practice guidelines (European Medicines Agency 2016). For interviews, focus groups, and co‐production workshops, consent will be obtained electronically prior to participation. For the online surveys, being anonymous at the point of entry, assumed consent is taken as participants will be required to confirm their understanding of the study's purpose, data usage, and storage via an introductory page before beginning the survey.

The patient information sheets were developed following the Health Research Authority (2024) guidance and templates and were reviewed by stakeholders, including men with IBD. The surveys, interview guides, and focus group frameworks were collaboratively designed and piloted with individuals affected by IBD and relevant stakeholders to ensure the questions are both appropriate and sensitive.

Interviews and focus groups will be facilitated by a registered nurse or an experienced qualitative researcher trained to address emotional distress and escalate concerns if necessary. If it is determined that an interview is negatively impacting a participant, it will be terminated. Additionally, men with IBD and their partners will receive a support leaflet with a curated list of resources to help manage depression, anxiety, and stress.

All study data will be managed in line with the General Data Protection Regulations (European Union Law 2016), the Data Protection Act (United Kingdom Public General Acts 2018), the ICH guideline for Good Clinical Practice E6(R2) (2016), and will comply with the standard operating procedures of REDACTED FOR PEER REVIEW (the study sponsor).

A collaboration agreement between the sponsor and higher education organisations supporting the study delivery has been established and approved by the funder. The full protocol is available at clinicaltrials.gov (Registration number: NCT06562751).

### 3.9 Rigour

Several strategies will be implemented at each stage of the research process to ensure scientific rigour (Creswell and Clark 2017). The content validity of the surveys has been ensured through conducting a thorough review of relevant literature and by consulting stakeholders, including men with IBD. Piloting of the surveys was undertaken to refine question clarity and ensure sensitivity to participants' experiences.

Trustworthiness has been assured using the criteria of credibility, transferability, dependability, and confirmability (Lincoln and Guba 1985). Credibility will be established by triangulating insights from men with IBD, the partners of men with IBD, healthcare professionals, and relevant stakeholders. This will be achieved through comparing and cross‐referencing the findings from the workstreams to identify points of convergence, divergence, and complementarity, producing a narrative synthesis (Popay et al. 2006) that informs the co‐development of the prototype intervention (Noyes et al. 2019). Researcher reflexivity will be used to further enhance the credibility of the findings and supported through the maintenance of a reflective journal, capturing key decisions and methodological considerations throughout the research process (Malterud 2001).

To enhance transferability, detailed descriptions of participant characteristics will be included to provide context. Dependability and confirmability will be ensured through the documentation of an audit trail, creating transparency in the research process and supporting the reproducibility of the study (Nowell et al. 2017).

The Standards for Reporting Qualitative Research (O'Brien et al. 2014) will be used to support the quality of reporting findings from this study. The reporting of this report has followed the SPIRIT guidelines (Chan et al. 2013).

### 3.10 Public and Patient Involvement

The study design and associated funding application was supported by a patient group and a patient co‐applicant. The patient group, which comprised UK‐based men across a diverse range of IBD diagnoses, age, sexualities, and ethnicities, was established with support from CCUK. The group met to discuss all aspects of the study design and support the ethics application process. Following the award of funding for the study, a stakeholder engagement group was formed comprising men with lived experiences of the disease, HCPs, and stakeholders. This group meets regularly and has supported the development of data collection methods, including surveys and interview guides, piloted data collection methods, and assisted with participant recruitment. The group will participate in the study delivery through to the dissemination stages.

### 3.11 Dissemination

A multi‐pronged dissemination strategy that extends beyond traditional academic audiences is essential to ensuring findings of this project are communicated widely to relevant clinicians, service‐user groups, and the public. The prototype intervention and any associated materials (e.g., evidence‐based summaries; information about the support that patients and partners need) will be distributed to IBD NHS services. Findings will also be shared with NHS service management networks, the Royal College of Nurses Gastroenterology network, and the British Society of Gastroenterology. Public‐accessible formats (e.g., self‐help materials; information sheets) will be provided to partner voluntary agencies (e.g., CCUK) and shared through social media, relevant podcasts, press releases, and blogs. It is intended that the findings will also be published in appropriate journals and presented at relevant conferences. An in‐person dissemination event in partnership with our stakeholders will be held at the end of the study.

The study protocol has been made publicly available on the sponsor's and the clinicaltrials.gov websites. It is intended that survey data will be made publicly available through an appropriate registry. As part of the consenting process, participants in the qualitative workstream will have provided consent for anonymised research data to be shared to support further research. However, the full qualitative dataset will not be made publicly available to safeguard the anonymity of participants and reduce the risk of interview discussions being interpreted outside of the context in which they were conducted.

## 4 Discussion

This protocol has described a mixed‐methods study aligned with phase 1 of the MRC framework for the development of complex interventions (Skivington et al. 2021). Data collected from the three UK surveys, semi‐structured interviews and focus groups will be analysed separately, then triangulated through a preliminary narrative synthesis (Rodgers et al. 2009) and cross‐validation to inform the co‐production workshops. The survey data will measure men's sexual health priorities and care preferences, supplemented by qualitative interview data that will provide in‐depth insights into the barriers faced by men when seeking and receiving sexual healthcare. The qualitative data will also support a better understanding of the nuanced ways in which sexual health assessments can be tailored to ensure they are disease‐ and gender sensitised. Through integrated interpretation of the qualitative and quantitative data, an enriched understanding of the most pressing needs of men with the disease, inclusive of members of underserved groups, such as those with non‐heterosexual orientations, will be developed. This will ensure that the co‐produced prototype intervention covers both the general sexual health priorities of men while also providing a clear pathway to benefit for those who may require highly specialised interventions. Cross‐referencing the data collected from men with the disease to the information provided by nurses and IBD services will help support the development of assessment prompts, information materials, educational resources, and pathways that are likely to be achievable, acceptable and useful to patients and healthcare practitioners. Aligning the workstreams to the MRC framework supports consideration of the varying healthcare systems likely involved in the delivery of the intervention, thus enhancing its feasibility and rapid implementation. Stakeholder engagement and patient involvement are integral to the study design, ensuring the creation of a prototype intervention that effectively addresses the disease‐and gender‐specific sexual health needs of men with IBD. By employing co‐production methods, the intervention will be tailored to be acceptable to diverse groups of men with the condition and feasible for implementation within existing NHS service frameworks.

The resulting prototype intervention will include a draft logic model that articulates the expected pathway to implementation. This model will outline inputs (e.g., resources), outputs (e.g., improved knowledge, attitudes, and behaviours), and anticipated outcomes (e.g., enhanced health status and quality of life). Based on preliminary work, candidate interventions are expected to include:

- A structured education/training programme for nurses to enhance confidence, preparedness, and best practices.
- An evidence‐based framework for clinicians to assess and manage men's sexual health effectively. This could involve a formal assessment tool and/or decision aids, communication models, visual aids, and discussion prompts.
- A gender‐sensitive patient information resource to support shared decision‐making between clinicians, patients, and their partners.

The use of co‐production will support the design of an intervention that is sensitive and relevant to the needs of men with IBD. Recently, clinical pressures within the UK NHS mean that people are currently waiting too long for a diagnosis or to receive specialist treatment, and there are insufficient staffing levels to meet patient need (Inflammatory Bowel Disease United Kingdom 2024). While the proposed intervention must meet the needs of both men with IBD and of IBD nurses, careful consideration will also be given to ensuring the prototype can be adapted to varying service structures and is feasible considering the barriers identified during the study.

### 4.1 Limitations and Risks to Study Delivery

There are some acknowledged limitations of the study design and possible risks to its delivery and processes. First, we used author‐designed surveys as no formally validated surveys exist. This may affect the reliability and generalisability of the results. Second, the success of the proposed intervention in addressing the needs of a diverse group of men with IBD depends on the recruitment of a broad and representative range of participants in workstreams 1 and 2. Due to the sensitive nature of the topic, it is anticipated that the recruitment of men with IBD and their partners may be a key challenge. In the IBD patient survey conducted in 2023, only 28% of respondents were male (Inflammatory Bowel Disease United Kingdom 2024). Likewise, ensuring adequate representation of men from black and Asian backgrounds with IBD will be difficult. To mitigate this risk, a recruitment strategy has been proposed that draws upon established networks to reach underrepresented groups. The advisory stakeholder group will also play a pivotal role in developing targeted strategies to ensure recruitment of a heterogeneous sample that is representative of the male IBD population.

The partners of people with IBD may have a role in helping those with the disease to adapt to the disease, and the involvement of partners in care has been suggested as a possible strategy for improving patient‐centred care (Dibley et al. 2014; Basson et al. 2010). This evidence, alongside feedback from the PPI group, led to the inclusion of partners in the semi‐structured interviews. However, it is recognised that challenges can occur when couples are interviewed together, such as one partner dominating the interview or producing a simplified ‘official’ account (Oliffe et al. 2014). In order to mitigate this problem in a sensitive and appropriate way, interviews will be offered jointly or individually, with the intention to undertake a proportion of interviews with the partners of men alone (determined by saturation and the number of joint interviews undertaken). Additionally, it is recognised that the recruitment strategy used for men with IBD may not reach their partners, and therefore the recruitment of men's partners may be reliant upon men with IBD distributing the study information. This could limit partner involvement and may not result in a representative sample of men's partners.

Recruitment of busy NHS clinicians may also pose a further key challenge. As with the recruitment of men with IBD, a multi‐pronged recruitment strategy and conservative time frames for recruitment should support the mitigation of this risk. The incentive of a £20 voucher for participation in the qualitative work may also support recruitment.

## 5 Conclusion

This proposed mixed‐methods study utilises nationwide cross‐sectional surveys, semi‐structured interviews, and asynchronous focus groups to address a key gap in the current evidence base. The findings of this study will help inform co‐production workshops with the aim of producing a prototype intervention that will provide nurses with improved understanding of the support, knowledge and skills required to effectively assess the sexual health needs of men with IBD. The overarching goal is to provide appropriate and patient‐centred care that has the potential to have important and immediate benefits for the quality of life of men with IBD and their partners, as well as acting as a catalyst for further research studies.

Dissemination of the findings beyond traditional academic audiences will be essential for establishing a pathway to patient benefit. A multi‐pronged evidence‐based dissemination strategy that reaches out to key stakeholders will be a priority, to ensure effective communication with clinicians, service‐user groups, and the public be the priority. This will include dissemination of materials for use within the UK NHS, including provision of resources for patients and their partners in accessible formats, such as written materials, infographics, and videos available online and via national patient organisations and groups. It is likely that a further evaluation of the prototype will be warranted, with likely progression to a trial in which the intervention will be compared to current care and economically evaluated.

## Conflicts of Interest

The authors declare no conflicts of interest.

## Supporting information


**Data S1:** jan70199‐sup‐0001‐DataS1.pdf.

## Data Availability

Data sharing not applicable to this article as no datasets were generated or analysed.
